# Where Does It Belong? Autonomous Object Mapping in Open-World Settings

**DOI:** 10.3389/frobt.2022.828732

**Published:** 2022-04-19

**Authors:** Edith Langer, Timothy Patten, Markus Vincze

**Affiliations:** ^1^ Vision for Robotics Laboratory, Automation and Control Institute, TU Wien, Vienna, Austria; ^2^ Robotics Institute, University of Technology Sydney, Sydney, NSW, Australia

**Keywords:** object detection, object matching, object mapping, open-world detection, autonomous robot, point-pair-features

## Abstract

Detecting changes such as moved, removed, or new objects is the essence for numerous indoor applications in robotics such as tidying-up, patrolling, and fetch/carry tasks. The problem is particularly challenging in open-world scenarios where novel objects may appear at any time. The main idea of this paper is to detect objects from partial 3D reconstructions of interesting areas in the environment. In our pipeline we first identify planes, consider clusters on top as objects, and compute their point-pair-features. They are used to match potential objects and categorize them robustly into static, moved, removed, and novel objects even in the presence of partial object reconstructions and clutter. Our approach dissolves heaps of objects without specific object knowledge, but only with the knowledge acquired from change detection. The evaluation is performed on real-world data that includes challenges affecting the quality of the reconstruction as a result of noisy input data. We present the novel dataset *ObChange* for quantitative evaluation, and we compare our method against a baseline using learning-based object detection. The results show that, even with a targeted training set, our approach outperforms the baseline for most test cases. Lastly, we also demonstrate our method’s effectiveness in real robot experiments.

## 1 Introduction

Industrial tasks such as fetching objects, mobile manipulation, patrolling, or supporting humans in robot assembly require an understanding of objects relevant for the task in relation to the environment. When asking people what they wish robots could do at home, cleaning, tidying up, and picking up items from the floor are top priorities (Bugmann and Copleston, 2011; Cakmak and Takayama, 2013; Bajones et al., 2018). While there are solutions for vacuuming or mowing the lawn, maintaining order is largely unsolved. To work towards the open challenges, several competitions have been started, for example, the ICRA 2018 “Tidy Up My Room” Challenge^1^ or the WRS RoboCup@Home (Okada et al., 2019) tidy-up task.

The tidy-up task is complex because a robot must operate in an unstructured and dynamic environment where it needs to localize known as well as unknown objects. To determine where objects belong to, the robot needs to have knowledge about the intended storage locations for these objects, e.g., in a knowledge base or another form of reference (Tenorth and Beetz, 2013). The focus of this paper is on the perception system for the tidy-up task and related applications, for which we refer to object detection and matching as the *object mapping* task.

Many approaches exist that detect objects in an environment by identifying changes between two visits based on camera data. A common method is to perform differencing between two single views using either color images (Furukawa et al., 2020; Sakurada et al., 2020) or RGB-D frames (Alimi et al., 2012; Mason and Marthi, 2012). Both modalities are affected by inaccuracies of view alignment. An alternative pathway is to reconstruct the environment, which has the benefit of providing a 3D object map to store object locations (Ambrus et al., 2014; Fehr et al., 2017). The major difficulty is to first create a consistent map and then align to this map at later visits given uncertainties in robot localization, view direction, or odometry. Options to handle this are to request users to give alignment cues (Finman et al., 2013) or assume sufficient robot accuracy (Björkman and Kragic, 2010; Ambrus et al., 2014; Song et al., 2015).

Object detection is the prerequisite to find matches. It reduces the object mapping task to the problem of comparing the locations of objects in the environment at two time instances. This definition is generic and independent of the specific robot task. For example, if the task is tidy up, a comparison is performed between the present situation and a *reference map*. If the task is patrolling, the observant robot will use all object detections to create a *present object map* for the new time instance. For fetching an object, the knowledge where this object was last seen, i.e., in the present object map, is used to retrieve it and, if not found, to start a search that may include information where the object has been found before.

Similarly to the object mapping task, the object rearrangement task as introduced by Batra et al. (Batra et al., 2020) also deals with the goal of transforming the current state of the environment into a target state assuming a closed world. This does not represent the real world that must consider objects that appear, and are therefore unknown, or disappear. Today most approaches assume a given and fixed set of objects, e.g., Bore et al. (2018) and Weihs et al. (2021). To develop more general methods, the task of open-world object detection is recently defined by Joseph et al. (2021). Objects from unknown classes need to be identified and then learned when label information becomes available. As a recent example, Kim et al. (2021) tackles the first aspect. They propose a method that generates class-agnostic object proposals in an open-world setting, but without classification. These approaches operate on small image patches and do not yet generalize to robotics applications in the 3D world.

Towards this goal, we present an approach that copes with all possible cases of static, moved, removed, and novel objects in different room settings. We partition a room into local horizontal surfaces, which is motivated by the fact that objects are typically found on furniture such as tables or shelves. Furthermore, it is infeasible for daily use to repeatedly and exhaustively scan an entire room. Tasks rather need to check if the object is at a specific location or surface. Finally, local surfaces can be easily extended to include other structures such as vertical surfaces to locate a broader variety of objects including pictures, switches, or door handles. This concept of local surfaces can be easily extended to multiple rooms. At the core of our approach is a comparison function to match detected objects to previously seen instances. To achieve this we represent surfaces where the objects reside as a 3D point cloud in the reference map. To autonomously create the surface partitioning, we exploit semantic segmentation. Finally, local surfaces enable high-quality reconstructions of every plane, which enhances the matching of detected objects using state-of-the-art methods such as Point Pair Features (PPF) (Drost et al., 2010). PPF is computationally cheap and runs on CPU only, which plays a considerable role for approaches running on mobile robots.

Our approach is evaluated on the *ObChange* (*Ob*ject *Change*) dataset, which extends prior work in Langer et al. (2020) with better local reconstructions. It encompasses multiple visits to five rooms with a total of 219 annotated objects. Taking all possible comparisons of visits per room into account, this leads to 961 objects for detection and matching. We report the results achieved on *ObChange* compared to a baseline using a learning-based detection approach as well as highlight possible failures and remaining challenges. Furthermore, we show the performance of our proposed approach using a fully autonomous system working in a real indoor environment.

To summarize, our contributions are:

• A procedure that uses semantic segmentation and surface fitting to reliably detect objects and that robustly handles all cases encompassed in an open-world, that is, static, moved, removed, and novel objects.
• An object mapping approach that does not rely on a trained classifier or pre-defined 3D models and, thus, works in an open-world setting by leveraging information extracted from a 3D representation.
• Presenting the *ObChange* dataset and an evaluation of different detection methods to categorize objects into the four cases.
• An evaluation on a fully autonomous robot that performs experiments in a real environment.

The remainder of the paper is organised as follows. Section 2 discusses related work for object mapping in open-world settings. Section 3 details our approach and Section 4 presents the experiments with *ObChange*, real experiments with a mobile robot, and a discussion of the results followed by the conclusion in Section 5.

## 2 Related Work

Object mapping and determining object relations across different time instances requires their detection and association. Our work focuses on the perceptual part involved for this task. Object detection in RGB images, as well as 3D data, is an active field in computer vision and robotics. While most of the single frame, learning-based detectors are limited by their training dataset, some methods working on 3D data use only geometric properties (Tateno et al., 2015; Furrer et al., 2018) or additional semantics (Grinvald et al., 2019). Although these methods are useful in open-world settings, this section reviews work related to object detection via scene differencing in 3D, which is closer to our approach as it also uses a reference map. Additionally, this section explores image-based methods that tackle the open-world assumption and discusses available datasets useful for the object mapping scenario.

For object change detection, *scene differencing* based on 3D data is a common approach (Finman et al., 2013; Ambrus et al., 2014; Fehr et al., 2017; Langer et al., 2017) because the only prerequisite are two aligned reconstructions of the environment. The methods deal with alignment inaccuracies and falsely detected objects by requesting human help (Finman et al., 2013), filter detected objects with morphological operations (Fehr et al., 2017; Langer et al., 2017), or limit the trajectory of the robot while creating 3D maps (Ambrus et al., 2014). Approaches based on scene differencing are in general not able to detect replaced objects.

Object detection based on *learning-based* approaches such as YOLO or Mask R-CNN are very popular, yet are limited to a closed world and show weakness by assigning unknown objects mistakenly a learned class with high confidence. Learning-based open-world object classification is an emerging research field (Pidhorskyi et al., 2018; Liu et al., 2019; Boccato et al., 2020; Perera et al., 2020), while the extension to open-world object detection is only recently defined (Joseph et al., 2021). Only few works exist for focusing on object detection, which deal with estimating uncertainty and therefore being able to distinguish unknown objects (Miller et al., 2018, 2019). Currently, they are not capable of gradually extending their knowledge when new classes emerge, which is essential to be useful in real-world applications. Incrementally extending the knowledge of a trained detector leads to the problem of catastrophic forgetting (French, 1999; Kirkpatrick et al., 2017), which is the challenge of maintaining robust performance on known classes as new classes are learned.

Only a few suitable *datasets* exist that not only support object change detection but also the categorization of different change types (novel, removed, etc.). Similarly, not many datasets provide information about object associations between two recordings. The datasets can be separated into synthetic frames-wise annotated datasets (Park et al., 2021; Weihs et al., 2021) and real-world datasets where the 3D map is annotated (Wald et al., 2019; Langer et al., 2020). Based on the task definitions from Batra et al.(Batra et al., 2020), Weihs et al. (Weihs et al., 2021) introduced a new dataset with object rearrangements in a virtual environment for studying how robots explore their environment. They set up two different versions of the rearrangement task. In the easier setting, the robot sees the current and the goal state at the same time, leading to perfectly aligned observations. In the advanced version, the robot must explore the environment in the target state first, and after the objects are moved, bring them back to their target location. A limitation of their task definition is that only objects present in the goal state can be out of place in the current state, thus assuming a closed world. ChangeSim (Park et al., 2021) is a synthetic dataset of warehouse scenes with different illumination and dusty air levels acquired with a drone. This is curated to support online detection approaches that work directly on frames. Therefore, first a correct pairing of frames from two different time instances must be found before change detection can be computed. For change detection, they define the following categories: new, removed, replace, rotated, or static. The most important category for object mapping, moved, is not defined. This makes it impossible to differentiate between a removed or a moved object. Wald et al. (Wald et al., 2019). introduce a real-world dataset acquired with a handheld device. Similar to Weihs et al. (Weihs et al., 2021), the dataset is designed for object instance re-localization. Unfortunately no novel or removed objects are considered. Another drawback is that mainly large items such as furniture are labeled and not objects that can be manipulated by a service robot. A real-word robotic dataset for change detection is acquired by Langer et al. (Langer et al., 2020). Objects that are labeled have been selected from the YCB object set (Calli et al., 2017). This dataset is only used for change detection and we extend it to provide the necessary reconstructions and ground truth for matching cases for open-world settings.

Only a few works exist that can be applied to the object mapping task. Bore et al. (Bore et al., 2018) detect objects based on the change detection approach of Ambrus et al. (Ambrus et al., 2014) and additionally track the movement of detected objects over time by defining a two-stage movement model, which is limited to a closed world. Also the baselines introduced by Weihs et al. (Weihs et al., 2021) work with a closed-world assumption, where at both time instances the same objects occur. The best performing method evaluated on their proposed dataset achieves a success rate of 8.2% when exploring the goal and current state subsequently and 17.9% when seen simultaneously. This is in line with the observation from Park et al. (2021), that pairing frames is a non-trivial task. It has to be noted that they propose an end-to-end learned approach for a robotic system without any knowledge of the environment. Finman et al. (Finman et al., 2013) discover objects through differencing of reconstructions. The focus of their work is on learning segmentation methods to re-discover objects in future visits, which are then used to segment the whole environment at the next visit. The result is used to find segments that overlap with the object. The biggest limitation of their work is the need of well aligned room reconstructions, where they rely on human input to define the overlapping parts between two reconstructions. This overlapping part is then aligned using ICP, which tends to fail if big parts in the overlapping area have changed. The approach closest to our work is by Song et al. (Song et al., 2015). By assuming that the robot stays in the same environment, their goal is to determine a global instance-based labeling and to further recognize individual objects. The method requires a high-quality reconstruction that is generated by an RGB-D camera array. Based on the full semantic labeling generated by a crowd-sourcing marketplace, objects are either classified as movable or non-movable. Non-movable objects are considered as background and used to align frames from different timestamps. For the remaining environment parts, a SIFT descriptor is computed and used for matching. Their approach heavily relies on the quality of manually labeled instance segmentation for the whole environment. In contrast, we propose a fully autonomous system using a surface concept to more efficiently partition an environment for different object mapping applications.

## 3 Object Mapping Using Local Surfaces for Matching

This section formally defines the problem of object mapping in arbitrary environments in Section 3.1. An overview of the perception components for this task is given in Section 3.2. Finally, the details for reconstruction, object detection, and object matching are described in Sections 3.3–3.5.

### 3.1 Problem Definition

The goal of this work is to detect objects in an environment and to further assign each object a category depicting its relationship to previous detections. The focus is on objects, which are detachable from the surface they are placed on and can be manipulated by a service robot. To remain task-independent, we compare objects present in an environment at time *t*_0_ and objects detected at a later time *t*_1_. We refer to objects detected at *t*_0_ as models and denote the set of detections as M . Objects detected at *t*_1_ are referred to as candidates and the set is denoted C . Detected objects are matched across the time instances, then categorized into static, moved, removed, and novel; see also Table 1. A static object is a candidate c∈C that has a matching model m∈M and where the distance between *c* and *m* is less than a threshold *d*. This threshold is selected depending on the uncertainty in robot localisation, the reconstruction, object detection, object placement, etc. The value may be different depending on specific applications. We use *d* = 20 cm throughout the paper. A moved object is a candidate that has a matching model but where the distance between the objects is greater than *d*. An object is considered removed if it exists in M but has no matching candidate in C . Novel objects are any candidates in C that have no matching model in M . The set of models M is the union of all static, moved, and removed objects while the intersection of these must be empty. Likewise, the set of candidates C is the union of static, moved, and novel objects while their intersection must be empty.

**TABLE 1:** Definition of the different categories an object gets assigned to when comparing an environment at two different timestamps.

Object category	Description
Static	Object did not move or only less than a distance *d* at time *t*_0_ compared to *t*_1_
Moved	Object is detected at time *t*_0_ and at time *t*_1_, but at different locations
Removed	Object is detected at time *t*_0_ but not at time *t*_1_
Novel	Object is detected at time *t*_1_ but not at time *t*_0_

### 3.2 System Overview

**FIGURE 1:**
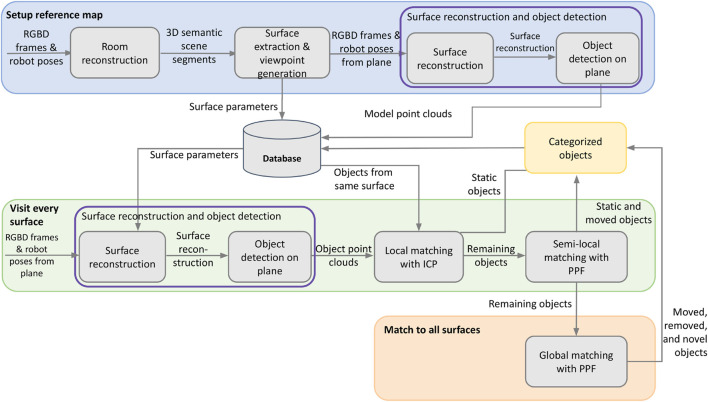
System overview of our approach. The setup of the reference is performed only once.

An overview of the proposed perception system for autonomous object change detection and mapping is given in Figure 1. The approach is composed of two phases: setup of the reference map (blue), which needs to be preformed only one time, and every run through the environment to visit all or a subset of surfaces (green and orange). If the larger structure of the room or the main surfaces (such as furniture) are significantly moved, phase one must be repeated to generate a new reference map.

This work leverages the concept of surfaces to focus object comparisons, which ultimately leads to improved object detection. Technically, this is achieved by first combining the reconstruction of the room and semantic segmentation to create a list of relevant surfaces; see Section 3.3. Then a more detailed scan of every surface is performed to improve the reconstruction. This high-quality reconstruction of each surface is used to detect objects (see Section 3.4), which are stored in a database as the reference object map for future change detection requests. When the robot revisits rooms and surfaces for its specific task, for example, tidying up a kitchen counter, a new detailed reconstruction is generated. Objects are extracted and then matched to those in the reference. The matching process is performed in three different ways to handle different cases as outlined in Section 3.5.

In this work we use only change as a cue for segmentation, which is fully applicable to open-world settings. If change occurs multiple times, then a heap of objects may become disentangled; otherwise, the heap will be considered as a single object. In the following sections, we refer to each detection as an object, both for single standing items or heaps of objects.

### 3.3 Reconstruction of the Indoor Environment and Plane Extraction

The first step for detecting objects is to identify the regions where objects are commonly located, in other words, the surfaces. Similar to our previous work (Langer et al., 2020), the search space for objects is reduced according to the assumption that objects are most often placed on horizontal planes in home environments (Björkman and Kragic, 2010; Marton et al., 2010). To extract horizontal planes, the environment is reconstructed using *Voxblox* (Oleynikova et al., 2017). This method runs on CPU only and is tightly coupled with ROS (Quigley et al., 2009), both great qualities when working with a robot. The coarse reconstruction of the environment, which is a result of the voxelized representation, is used to geometrically search for planes. To do so, the reconstruction is transformed into a point cloud by extracting the centroids of all voxels. In addition, *SparseConvnet* (Graham et al., 2018) is applied to the reconstruction to retrieve a semantic label of each point and consequently to exclude non-relevant regions for the plane search; all points are removed that are not assigned any of the following classes: cabinet, bed, chair, sofa, table, bookshelf, counter, desk, shelves, nightstand, other props, other structure, and other furniture. Since we focus on horizontal planes, only points with a normal facing upward are retained. Finally, for each semantic class the remaining points are downsampled and input to RANSAC (Fischler and Bolles, 1981) to fit to a plane. Each iteration generates one plane and these points are removed from the input to enable further plane fitting. The loop ends when the extracted plane consists of less than a certain number of points.

For each plane, descriptive information such as the plane coefficients, convex hull points, and centroid are stored in a database. Additionally, waypoints for the robot to navigate to when inspecting the plane are computed. Waypoints are equally distributed positions around the plane at a fixed distance to the edge of the convex hull. The pose of each waypoint is described by its position and an orientation that faces the center of the plane. All the surface information is used for subsequent visits.

### 3.4 Reconstruction of the Surface and Object Detection

**FIGURE 2:**
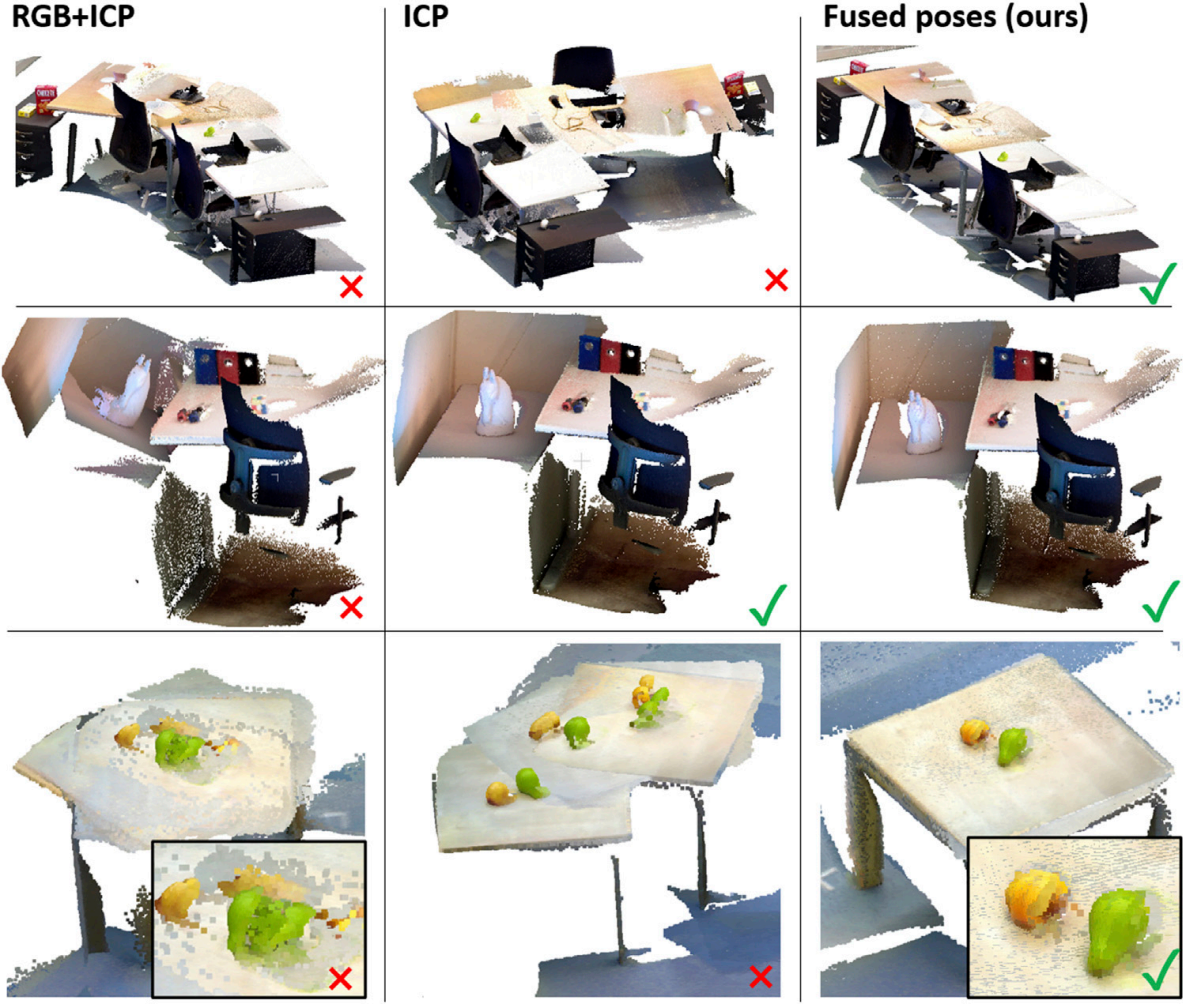
Each row shows a surface of one of the environments in our dataset. The first column is the result of ElasticFusion using RGB and ICP information to estimate the camera pose, the second row shows results using only ICP, and the last column shows the result of fusing the robot poses with the estimated camera poses from ElasticFusion (only ICP).

Once the global reconstruction of the environment is created, a higher-quality local reconstruction is generated for each surface to enable more precise object detection. In this work we use *ElasticFusion* (Whelan et al., 2015) for the local surface reconstruction. It uses both photometric and geometric pose estimation, which is configured using a relative ICP/RGB tracking weight parameter. While *ElasticFusion* is more precise than *Voxblox*, it still suffers specific failure cases that need to be addressed for robust operation on a mobile robot. Firstly, viewpoints focusing on large planar and low-textured surfaces have too few features to track the camera pose, which results in misalignment (see Figure 2 [top]). Secondly, changing lighting conditions resulting in over- and underexposured images is problematic for registration as can be seen in Figure 2 (middle). Another source of error are geometric symmetries as well as low depth disparity. Figure 2 (bottom) shows an example reconstruction using RGB and ICP registration but suffers from duplicated and misaligned objects or smeared objects.

To countermeasure those real-world problems, we propose a computationally simple solution. Our idea is to assist the tracking method whenever the estimated trajectory begins to significantly diverge from the reported robot odometry data. Clearly, odometry data has inaccuracies; thus, using it directly is not sensible. Whenever the registration fails completely (e.g., Figure 2 [top]) the estimated pose and the odometry pose differ significantly. Resetting the estimated pose to the odometry data is not feasible either because this can lead to smeared reconstructions. Our approach is to blend the poses from the camera tracking and odometry data to repair drift and misalignment error in the running reconstruction. For each frame, before running *ElasticFusion*’s frame processing, we recompute the pose given the last estimated pose and the robot pose from odometry data. To this end we first compute the difference between the last estimated pose *E* and the current robot pose *P*.

More specifically, the poses are represented by transformation matrices:

$$E=\left[R_{E}|t_{E}\right],P=\left[R_{P}|t_{P}\right]\in SE_{3}$$ (1)

with rotation components $R_{E},R_{P}\in SO_{3}$ and translation components $t_{E},t_{P}\in R^{3}$ . Next the matrix is computed:

$$D=P^{-1}E=\left[R_{D}|t_{D}\right].$$ (2)

Given this, we compute the angular difference:

$$\phi =|\arccos\left(\frac{\text{trace}\left(R_{D}\right)-1}{2}\right)|,$$ (3)

to derive the mixing term:

$$\lambda =\text{max}\left(\text{min}\left(1.0,{\left(\frac{\phi }{z_{r}}\right)}^{2}\right),\text{min}\left(1.0,{\left(\frac{\left|t_{D}\right|}{z_{t}}\right)}^{2}\right)\right),$$ (4)

where *z*_*r*_ and *z*_*t*_ are constant scaling factors. This allows the modified *E*′ to be used as replacement for *E* through a linear combination of the poses:

$$E^{\prime }=\lambda P+\left(1-\lambda \right)E.$$ (5)

The more the two poses disagree (i.e., the estimated pose diverges from the pose measured by the robot), the more the odometry pose is used in hope for future agreement. Clearly, if odometry is less accurate, the results will degrade. However, it still prevents ElasticFusion from completely failing in difficult scenes. In our experiments we found good results with *z*_*r*_ = 0.2 and *z*_*t*_ = 0.2, which is used for all experiments.

Integrating the tracked camera and robot poses generates high-quality reconstructions in real-world scenarios from the robot trajectory. This adaption is used to create a reconstruction for each extracted plane that is stored in the database before each is processed separately. For each plane, its parameters and waypoints derived in the setup stage are queried from the database. Based on this information, the robot navigates around the plane while the camera is directed to the center of the plane. Before *ElasticFusion* transforms the camera stream into a reconstruction, the depth images are pre-processed by cropping them such that only the plane is retained. Cropping the depth input prevents *ElasticFusion* from trying to align the background (e.g., walls) at the expense of reconstruction accuracy of objects placed on the plane. Benefiting from the local surface concept, trajectories to create plane reconstructions are comparably short and result in precise reconstructions. From these generated plane reconstructions, objects are extracted by removing the points of the plane according to the known plane parameters. The parameters *α*_max_, to allow deviation from a perfect horizontal plane, and *d*_*plane*_, which defines the maximum distance of inlier points to the detected plane, have to be chosen to take into account small inaccuracies. The remaining points within the convex hull are clustered using Euclidean distance. All points up to 0.3 m above the plane are considered; however, this value can be chosen depending on the application. We use a minimally shrinked convex hull to reduce the number of false positive detections such as walls or arm rests. At this stage, we do not try to separate objects in clutter and treat each cluster as an object.

### 3.5 Object Matching and Categorization

To support high-level robot tasks, objects are assigned one of the four categories, which is an indicator for what action should be performed with the object. For example, a static object should be left alone while a moved object should be returned to its original location. Performing the categorization requires the determination of which objects are present in different visits and additionally finding those that are matched. The following subsections explain the different stages of matching, which are also depicted in Figure 1.

#### 3.5.1 Local Matching

For each detected object, a check is performed to determine if it is still approximately located at the same position. If there exists a model and a candidate within a distance less than *d*, they are considered a potential match. A confidence score for the match is computed by aligning their point clouds with ICP, which is suitable in this case as their close proximity provides a good initial registration. Two scores are then computed if ICP converges: one for the model *S*_*m*_ and one for the candidate *S*_*c*_ due to the object potentially not being symmetric. The model and the candidate are a match if min (*S*_*m*_, *S*_*c*_) > *τ*_*icp*_ for a given threshold *τ*_*icp*_.

Formally, we consider the model point cloud $P_{m}$ and candidate point cloud $P_{c}$ . For each point $p_{m}\in P_{m}$ , a set of points $Q_{m,c}\subset P_{c}$ is determined as the collection of all corresponding points in $P_{c}$ that have a distance to **p**_*m*_ less than the inlier threshold *τ* after alignment. A score is then computed for **p**_*m*_ and each $p_{c}\in Q_{m,c}$ , which is composed of the geometric and color similarity. Given the point normals, **n**_*m*_ and **n**_*c*_, the geometric score is given by:

$$s_{geo}=\left\{\begin{array}{cc} n_{m}\cdot n_{c}\; & \text{if }n_{m}\cdot n_{c}\geq \tau_{geo},\\ 0\; & \text{otherwise}. \end{array}\right.$$ (6)

The color score is computed as:

$$s_{col}=\left\{\begin{array}{cc} 0\; & \text{if }\kappa_{m}⊖\kappa_{c}\geq \tau_{col}\\ 1-\frac{\kappa_{m}⊖\kappa_{c}}{\tau_{col}}\; & \text{otherwise}, \end{array}\right.$$ (7)

where ***κ***_*m*_ and ***κ***_*c*_ are the color values of the points **p**_*m*_ and **p**_*c*_ in LAB-space and ⊖ is the CIEDE2000 color difference (Luo et al., 2001). *τ*_*geo*_ and *τ*_*col*_ are thresholds. The similarity score between **p**_*m*_ and **p**_*c*_ in the correspondence set is the weighted combination of the geometric and color scores:

$$s_{m,c}=\left\{\begin{array}{cc} 0\; & \text{if }s_{geo}=0\vee s_{col}=0\\ ws_{geo}+\left(1-w\right)s_{col}\; & \text{otherwise}, \end{array}\right.$$ (8)

where w balances the contribution of the geometric and color similarity.

The overall fitness score for the model is defined as:

$$S_{m}=\frac{1}{\left|P_{m}\right|}\underset{p_{m}\in P_{m}}{\sum }s_{m,c}^{*},$$ (9)

where $s_{m,c}^{*}$ is the best similarity score for **p**_*m*_ given all the scores computed for the points in its correspondence set $Q_{m,c}$ . Likewise, the overall fitness score for the candidate is computed:

$$S_{c}=\frac{1}{\left|P_{c}\right|}\underset{p_{c}\in P_{c}}{\sum }s_{c,m}^{*}.$$ (10)

A match is recorded if at least one of the scores *S*_*m*_ or *S*_*c*_ is greater than the matching threshold *τ*_*icp*_. If only a subset of the points match, it is necessary to examine if either or both of the model and candidate need to be split into independent objects. A split based on the overlapping points is insufficient due to possible over or under segmentation of the objects. Therefore, to generate more precise object boundaries we perform region growing based on color and normal similarity where only points that contributed to the score are seed points. For all seed points, points within a certain radius are added if color and normal are similar enough compared to the seed point. The maximum allowed color difference *rg*_*col*_ is computed with the CIEDE2000 formula using the color values in the LAB-space of both points. The dot product from the normal vectors is used to check if the angle difference is not greater than *rg*_*geo*_. Points that fulfil both criteria are added and act as new seed points. If no more points can be added, the region growing stops. All points in the result that belong to the model or candidate are categorized as static. The remaining points form a new object in the respective set.

#### 3.5.2 Semi-local Matching

ICP alignment detects static objects in case of close proximity between the two instances. For other objects in the environment, where the initialization is poor, a more robust matching scheme is required that is independent of the pre-alignment. We propose to match objects by using the PPF global descriptor. PPF is a simple learning-free descriptor, nevertheless top-ranked in pose estimation challenges (Hodaň et al., 2018). In our system, PPF is computed for each unmatched object and the pipeline returns for each candidate between zero and ten hypotheses for each model. For each hypothesis we compute a confidence score for the model and the candidate as formulated in Eq. 9 and Eq. 10 and then compute the average. Only the best fitting hypothesis from each model is retained per candidate. Note that planar and small objects are filtered before applying PPF matching. All objects are downsampled to achieve a unified point density with a voxel size *v* and only objects with more than *obj*_min_ points and where less than *τ*_*plane*_ of the points can be explained by a plane model are kept. Otherwise their geometric characteristic is too generic and would result in many false matches.

More concretely, the matching problem is simplified by eliminating objects that match with a high certainty. To unravel the hypothesis we use a bipartite maximum matching graph (Edmonds, 1965). The nodes on one side are the models and on the other side the candidates. A connecting edge exists if the model hypothesis for a candidate fulfils min (*S*_*m*_, *S*_*c*_) > *τ*_*high*_, where *τ*_*high*_ is a fixed threshold. The weight of an edge is the average of the two scores. The maximum weighted matching of the graph is then computed. For each model-candidate pair in the graph solution we compute the spatial distance and categorize it either as static or moved. These models and candidates are considered as fully matched and are not processed any further.

With the reduced set of models and candidates, a new graph is built where the condition for an edge is relaxed. An edge is created if min (*S*_*m*_, *S*_*c*_) > *τ*_min_ and avg (*S*_*m*_, *S*_*c*_) > *τ*_*low*_. With *τ*_*low*_ ≪ *τ*_*high*_ it is possible to match models and candidates in heaps. In such cases it is often infeasible to achieve a very high confidence because of objects that are clustered together and increase the number of points used to normalize the score. This concept also helps to overcome deficits arising from incomplete reconstructions caused, for example, by few viewpoints or occlusions. The extracted matching results from the graph are then processed the same way as described in Section 3.5.1: starting from the matched points region growing is performed to extract all points from the reconstruction belonging to the matched model/candidate. These points are then categorized as static or moved depending on their spatial distance. Remaining points are considered as an additional model/candidate.

For all unmatched candidates, new hypotheses with the unmatched models are computed. The matching process restarts by building a graph with the relaxed edge condition. This procedure is repeated until no more matches are found.

#### 3.5.3 Global Matching

The final matching procedure considers objects that have been moved between different surfaces. This is performed by collecting all models and candidates from all surfaces that were not matched in the local or semi-local checks. Technically, the same approach as described in Section 3.5.2 is applied but now all objects from all surfaces are pooled together to perform global matching. The PPF descriptor is the basis for hypotheses creation and computed confidence scores are used as edge weights for a maximum weight bipartite graph. In the case that there is no match for a candidate or a model in the entire environment, the candidate is considered new or a model is considered removed.

**FIGURE 3:**
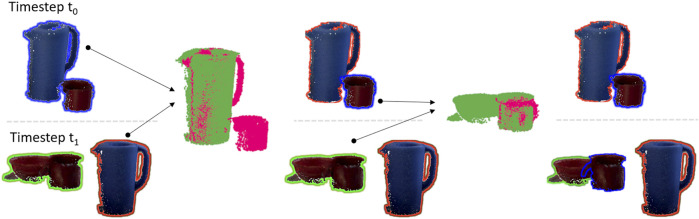
Example showing how objects are separated using PPF matching. Objects next to each other having the same shadow color are recognized as one object by the robot. After objects are matched they have the same shadow color in both recordings.

The advantage of our approach is that the robot does not need to learn object models in a cumbersome process, but inherently extends its knowledge through change detection. Figure 3 shows an example of the clutter dissolving capabilities of our system on an example from the real-world dataset. At timestep *t*_0_ a pitcher and a mug are detected next to each other. They are treated as one object. At timestep *t*_1_ the pitcher is a single standing object, while the mug belongs to a pile together with the bowl. First the pitcher is matched and with that information the mug is separated from the pitcher in *t*_0_. Now the separated mug is matched with the mug in *t*_1_. The result of the matching process is that all heaps are disentangled and instead of three object clusters the robot is now aware of five individual objects.

## 4 Experiments

This section evaluates the performance of our approach for detecting and categorizing object changes. It is compared to two variants of a learning-based method as baselines and quantitative experiments are conducted with the newly created *ObChange* dataset, consisting of recordings from a real autonomous robot (see Section 4.1). Additionally, we qualitatively demonstrate the applicability of our method in real-world scenarios with online experiments using a real robot (see Section 4.2). Finally, we discuss the indication of our experimental results and the consequent open research questions in Section 4.3. For reproducibility we give an overview of all parameters used for the dataset and real-world evaluation in Table 2.

**TABLE 2:** Parameters used for evaluation.

Method	Parameter	Value
Local plane extraction	Maximum angle *α*_max_ between plane normal and upward-directed axis	5°
Local plane extraction	Inlier distance threshold *d*_*plane*_ for plane model	0.015 m
Matching score	Inlier threshold *τ* for radius search	0.01 m
Matching score	Color threshold for point-wise matching *τ*_*col*_	20
Matching score	Dot product threshold between two normal vecots *τ*_*geo*_ for point-wise matching	0.95
Matching score	Linear weight factor ω for combined score	0.7
Object filtering	Voxel size *v* for object downsampling	0.005 m
Object filtering	Minimum number of object points *obj*_min_	200
Object filtering	Proportion of object points to count as plane *τ*_*plane*_	0.9
Region growing	Point inlier radius *r*	0.01 m
Region growing	Maximum allowed angle between normal vectors of neighboring points *rg*_*geo*_	5°
Region growing	Maximum allowed color difference between neighboring points *rg*_*col*_	15
Local matching	Minimum score for candidate match *τ*_*icp*_	0.7
Semi-local matching	Low score threshold *τ*_*low*_ for graph edge	0.4
Semi-local matching	High score threshold *τ*_*high*_ for graph edge	0.8
Semi-local matching	Minimum score for model and candidate *τ*_min_	0.2
PPF	Distance sampling rate as defined in Drost et al. (2010)	0.025
PPF	Orientation sampling rate as defined in Drost et al. (2010)	5

### 4.1 Evaluation on the Robotic Dataset ObChange

Since no suitable dataset to evaluate object mapping exists, we extend the dataset from Langer et al. (2020). Its description is given in Section 4.1.1. Section 4.1.2 introduces the metrics to measure the performance for the evaluation. In order to analyze our approach quantitatively, we compare it to two learning-based baseline using state-of-the-art methods as outlined in Section 4.1.3. In Section 4.1.4 we show overall performance and discuss in detail the results and open challenges in Section 4.1.5.

#### 4.1.1 The ObChange Dataset

For the quantitative evaluation of the object mapping task, we create the *ObChange* dataset, which consists of RGB and depth images acquired with the onboard Asus camera mounted on the head of the Human Support Robot from Toyota (Yamamoto et al., 2019). Additionally, the transformation matrices between different coordinate frames are recorded. The dataset is recorded for five different rooms or parts of rooms, namely a big room, a small room, a living area, a kitchen counter, and an office desk. For each room a version in a clean state exists that is used to extract surfaces. Each room is visited by the robot at least five times while between each run a subset of objects from the YCB dataset (Calli et al., 2017) is re-arranged in the room. For each setup between three and 17 objects are placed. Furthermore, furniture and permanent background objects are slightly rearranged compared to the reference. These rearrangements are small so that the moved furniture does not interfere with the robot’s navigation and that the moved objects are considered irrelevant in a tidy-up task. We use all rooms and all different recordings, which include YCB objects, in total 26 recordings.

The *ObChange* dataset extension from the data used in Langer et al. (2020) is for the reconstructions necessary for object matching. For each room a 3D semantically labeled reconstruction is created, which is used to identify horizontal planes as described in Section 3.3. For each detected surface, images from the recorded stream where the surface is visible are extracted, depth images are masked according to the plane parameters, and *ElasticFusion* is used to reconstruct the area. Only with the combination of odometry pose and camera tracking pose as in Eq. 4, suitable reconstructions for all surfaces are achieved. In this dataset, the robot drives exhaustively through an environment, leading to surfaces of interest being seen several times. Unfortunately, *ElasticFusion* cannot handle non-continuous input data. To overcome this problem, we create several reconstructions and merge them using ICP. We visually check the results and have adjusted them manually if needed. This manual step is not needed in the real world where the robot moves around the surface only once. The collection of all surface reconstructions together with their point-wise labeling of all YCB objects form the *ObChange* dataset. It is available at https://doi.org/10.48436/y3ggy-hxp10.

Compared to our previous work (Langer et al., 2020) we are not only interested in detecting all objects, but also to assign them to one of the four categories: static, moved, removed, and novel. To create a more meaningful evaluation of the possible categories, we compare each recording with all other recordings of the same room, leading to 961 objects in total. For each comparison the objects of both recordings are counted, meaning that static and moved objects are counted at *t*_0_ and *t*_1_. Table 3 gives an overview of the data for each room.

**TABLE 3:** Overview of the dataset used for the quantitative evaluation. Except for the first two columns the numbers state the sum over all possible comparisons per room.

Rooms	#Visits	#Surfaces	#Objects	#Static	#Moved	#Removed	#Novel
Big Room	6	12	425	28	260	50	87
Small Room	5	5	208	72	62	25	49
Living Area	5	11	108	0	54	30	24
Office Desk	5	4	100	12	28	24	36
Kitchen Counter	5	2	120	10	66	14	30
	26	34	961	122	470	143	226

#### 4.1.2 Metrics

In *ObChange* only YCB objects change, i.e., are novel, moved, or removed. All other objects are static and therefore irrelevant for change detection. Given that, we apply the following metrics:

• *Detected objects*: The number and percentage of detected YCB objects on surfaces.
• *Correctly categorized objects*: The number and percentage of YCB objects that are correctly categorized as static, moved, removed, or novel.
• *False positives*: The number of background objects that are not categorized as static plus the number of objects that are correctly classified, but parts of it have an incorrect category assigned.

#### 4.1.3 Baseline

To the best of our knowledge, no other work exists to categorize objects into static, moved, removed, and novel by comparing two time instances. Thus, in order to compare our approach for solving object mapping, we extend the recent work of Oliveira et al. (de Oliveira et al., 2021) for the task. Their proposed method detects objects with Yolov3 (Redmon and Farhadi, 2018) trained on the COCO dataset (Lin et al., 2014) in each frame. These object detections are used to create a 3D object map by temporal and spatial associations. We utilize this 3D object map to perform object matching. Thus, based on their work we develop two baselines. Instead of Yolov3 we use Mask R-CNN(He et al., 2017), another state-of-the-art object detector, because it is not only readily available for the established COCO dataset but also for the YCBV dataset (Xiang et al., 2017). We refer to the baselines as *COCO-baseline* and *YCBV-baseline*.

**FIGURE 4:**
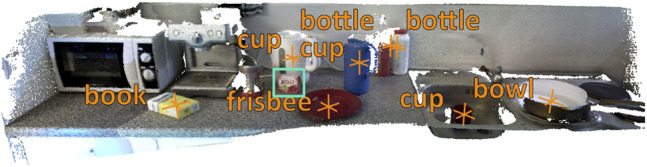
3D object map of detected objects using Mask R-CNN trained on COCO created with the approach from de Oliveira et al. (2021). Besides some background objects, all YCB objects are detected, except for the gelatin box (marked with cyan rectangle).

For both baselines we create 3D object maps for all dataset recordings using the standard parameters mentioned in de Oliveira et al. (2021) with the following exceptions: (1) use Mask R-CNN instead of Yolov3, (2) decrease the distance threshold for the bounding box center distance to 0.0001 (which equals to 30 pixels) and decrease the spatial association to 0.3 m for better results. The RGB images from the recordings and the estimated poses from *ElasticFusion*, which are more accurate than pure robot poses, constitute the input for 3D object maps. For the *COCO-baseline* we do not include detected objects from the following COCO categories in the 3D object map because they cover classes not relevant for indoor object change detection: person, vehicle, outdoor, animal, furniture (except potted plant), and appliance. The example in Figure 4 shows the detected objects with their assigned classes overlayed on the reconstruction.

Based on the generated 3D object maps, we compute the changes between two visits to a room using the assigned labels from the object detector. An object with a class label that exists in the visit at time *t*_0_, but not at time *t*_1_, is considered removed and vice versa as novel. If there is exactly one object of a specific object class in both visits, it is either categorized as static or moved depending on the distance between the object’s bounding box centers compared to the threshold *d*. In the case that several objects from the same class are detected, we find an association by utilizing the feature vector of the second last layer of Mask R-CNN. This is inspired by Qiu et al. (Qiu et al., 2020) who extract the features from the last layer as instance-level features. However, in our experience features from the last layer are already too class-specific, whereas the second last layer is more suitable for instance comparisons. Because an object is usually detected in several frames, the feature vector is extracted for each. To find the best matching instances from the same class within two visits, we compute for each object at *t*_0_,the dot-product between all its feature vectors with all feature vectors of all objects from *t*_1_, then match the object that achieves the highest value. Depending on the distance between the two matched objects, they are either categorized as static or moved.

The baseline using Mask R-CNN trained on the YCBV dataset (Xiang et al., 2017) is evaluated to analyze the performance when provided a tailored training set in comparison to the more general COCO dataset. Park et al. (Park et al., 2019) provide the weights for Mask R-CNN, which they trained for their pipeline to participate in the BOP challenge (Hodaň et al., 2018) on the YCBV dataset.

#### 4.1.4 Evaluation

Our evaluation is based on manually labeled data. In *ObChange* all YCB objects are point-wise labeled in all plane reconstructions for all the recordings. Besides the object point indices, the object name is stored as ground truth data. The categories (static, moved, removed, novel) are extracted given two recordings and the ground truth: If the object name exists only in one of the two recordings, the object is novel or removed. If it occurs in both recordings, we compute the centroid of both objects and, depending on the Euclidean distance, assign the category static or moved.

Our method works directly on the plane reconstructions and therefore the resulting object points match directly to the labeled data, and no further processing is needed. We consider an object as detected if at least 50% of the points in the result overlap with the labeled points. Each point in the result has one of the four categories assigned. The object matching stage may erroneously assign different labels to data points from the same object due to imprecise region growing. Therefore, we use the maximum voted category per object and compare it against the ground truth. If the categories match, the object is correctly categorized. Otherwise, it counts as a wrongly categorized object. For the categories static and moved, the correct association of the two involved objects must be given to be counted as correct. For example, it is not enough that an object at time *t*_0_ and *t*_1_ is categorized as moved; also, the association that the object at *t*_0_ moved to the location of the object at *t*_1_ must be given. The sum of false positives combines two different failure cases: (1) a YCB object where parts of it are wrongly classified. An object is therefore counted as correctly classified and at the same time it is a false positive. (2) Points in the result that are categorized as moved, removed, or novel but do not belong to any YCB object are from a static background object. We cluster the points and each cluster is counted as one false positive object. Equally to Langer et al. (2020), this metric is an approximation because no ground truth labeling for background objects exists.

Both baselines, in contrast, cannot be evaluated point-wise. Each detected object is described by a single 3D location and a label. Therefore, for each object in the ground truth, the centroid is computed and the closest object in the result is identified. If the distance is less than 10 cm, we count it as a detected object; otherwise, it is a false positive. Further, we check for each detected object if there is a nearby ground truth object with the same category, which was not already matched. If so, the detected object is correctly categorized. The associations for moved and static detected objects must correspond to the ground truth. For the evaluation of the *YCBV-baseline*, we remove objects from the ground truth that do not appear in the YCBV dataset and are therefore not used for training.

We provide an additional evaluation based on the fact that if a static or moved model-candidate pair is not detected, a subsequent failure may occur. For example, if a moved mug is detected at *t*_0_, but not at *t*_1_, it can never be categorized correctly as moved. Therefore, we re-evaluate the methods based on an adapted ground truth. Only detected objects are considered when re-computing the categorization for the ground truth objects. The correct categorization for the mug in the previously mentioned example would then be *removed*. This way we evaluate the categorization process stand-alone and independent of the preceding detection performance.

#### 4.1.5 Results

A summary of the performance on *ObChange* of our proposed method as well as the two baselines is given in Table 4. Our method detects 91.8% of all the labeled objects in the dataset while achieving the lowest number of false positive objects. Inspecting the detection rates of the two baselines, the result is surprising. Applying Mask R-CNN trained on the COCO dataset outperforms Mask R-CNN trained on the YCBV dataset by a significant margin although the ground truth objects in *ObChange* are selected from the YCB objects. Recently, Dhamija et al. (Dhamija et al., 2020) investigated state-of-the-art object detectors and their performance in open-world settings. They showed that all methods detect objects from classes not presented during training with high confidence, despite the fact that object detectors should only detect objects from known classes. This could explain the good performance of *COCO-baseline*, although trained on different classes. We conjecture that the detection performance of *YCBV-baseline* is only about 43% because of two reasons: (1) although the objects used in *ObChange* are from the official YCB object set, some have a slightly different texture than the objects used in the YCBV dataset and (2) the training data is captured from a certain distance range, while the robot did not always get as close to the objects in the dataset. Therefore, we assume that the object detector overfits to the training data, which is a huge problem when applying it to images that are dissimilar to the training dataset. The detection rate could be increased by using a lower confidence threshold; however, this would also increase the number of false positives.

**TABLE 4:** Results of the baseline trained on COCO and YCBV compared to the results of our method evaluated on *ObChange* and averaged per room.

Result using Mask R-CNN trained on COCO dataset					
Rooms	#Total objects	#Detected objects	#Correctly categorized	#Correctly categorized in detected objects	#False positives
Big Room	425	340 (80.0%)	117 (27.5%)	130 (38.2%)	218
Small Room	208	164 (78.8%)	102 (49.0%)	106 (64.6%)	10
Living Area	108	72 (66.7%)	61 (56.5%)	65 (90.3%)	20
Office Desk	100	64 (64.0%)	50 (50.0%)	55 (85.9%)	64
Kitchen Counter	120	88 (73.3%)	50 (41.7%)	57 (64.8%)	22
Overall Performance	961	728 (75.8%)	380 (39.5%)	413 (56.7%)	334
**Result using Mask R-CNN trained on YCBV dataset**					
** Rooms**	**#Total objects**	**#Detected objects**	**#Correctly categorized**	**#Correctly categorized in detected objects**	**#False positives**
Big Room	215	95 (44.2%)	48 (22.3%)	75 (78.9)	219
Small Room	100	32 (32.0%)	14 (14.0%)	18 (56.3)	75
Living Area	56	20 (35.7%)	12 (21.4%)	18 (90.0)	22
Office Desk	40	24 (60.0%)	18 (45.0%)	24 (100)	36
Kitchen Counter	100	48 (48.0%)	24 (24.0%)	34 (70.8)	49
Overall Performance	511	219 (42.9%)	116 (22.7%)	**169 (77.2%)**	401
**Result of our approach**					
** Rooms**	**#Total objects**	**#Detected objects**	**#Correctly categorized**	**#Correctly categorized in detected objects**	**#False positives**
Big Room	425	419 (98.6%)	286 (67.3%)	290 (69.2%)	66
Small Room	208	183 (88.0%)	131 (63.0%)	136 (71.6%)	13
Living Area	108	92 (85.2%)	75 (69.4%)	78 (84.48%)	10
Office Desk	100	88 (88.0%)	76 (76.0%)	78 (88.6%)	24
Kitchen Counter	120	100 (83.3%)	49 (40.8%)	56 (56.0%)	28
Overall Performance	961	**882 (91.8%)**	**617 (64.2%)**	638 (72.3%)	**141**

From the detected objects 64.3% are correctly classified with our approach. This is 1.5× more than *COCO-baseline* and 3× more than *YCB-baseline*. The difference between the results evaluated on the original ground truth and the adapted ground truth (*#Correctly categorized in detected objects*) is small for our approach because we achieve a high detection rate. For *YCBV-baseline*, the difference is significant. Comparing the adapted ground truth, *YCBV-baseline* slightly outperforms our approach in terms of correctly categorized objects (given that a substantially lower number of objects is detected in the first place). However, it has significantly more false positives even though it is specifically trained for the objects in the dataset.

**FIGURE 5:**
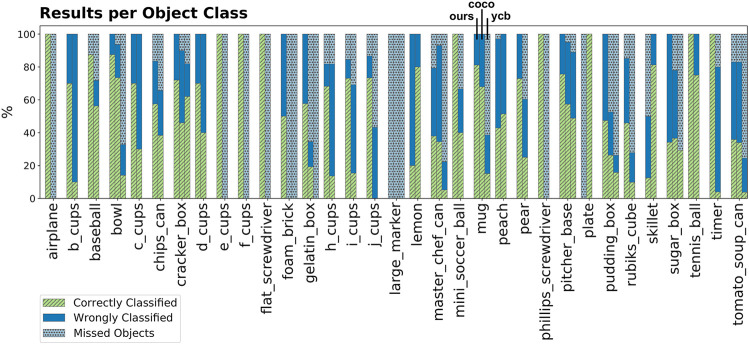
Overview of YCB objects used in the dataset. The performance for each object is shown for our approach (first bar), *COCO-baseline* (second bar) and *YCBV-baseline* (third bar). If the object is not in the YCBV dataset, no bar for *YCBV-baseline* is visualized.

The performance for each YCB object used in the evaluation is shown in Figure 5 for our approach and the baselines. It shows that the smallest object in the dataset, the large marker, cannot be localized by any method. However, our method was able to localize other small objects such as the screwdrivers. It can be seen that our method has difficulties detecting a plate because many points can be explained by the supporting plane model and are within the distance threshold *d*_*plane*_. Interestingly, for all objects from the YCB dataset the localization performance of *COCO-baseline* is superior compared to *YCBV-baseline*. The lemon is the only object where our approach performs significantly worse in categorizing than the baseline. The reason is that the lemon is small as well as part of a pile in most scenes. The wrong categorizations of the master chef can are the result of incomplete object reconstructions and the confusion with the pitcher, which has very similar appearance.

### 4.2 Robot Experiments

**FIGURE 6:**
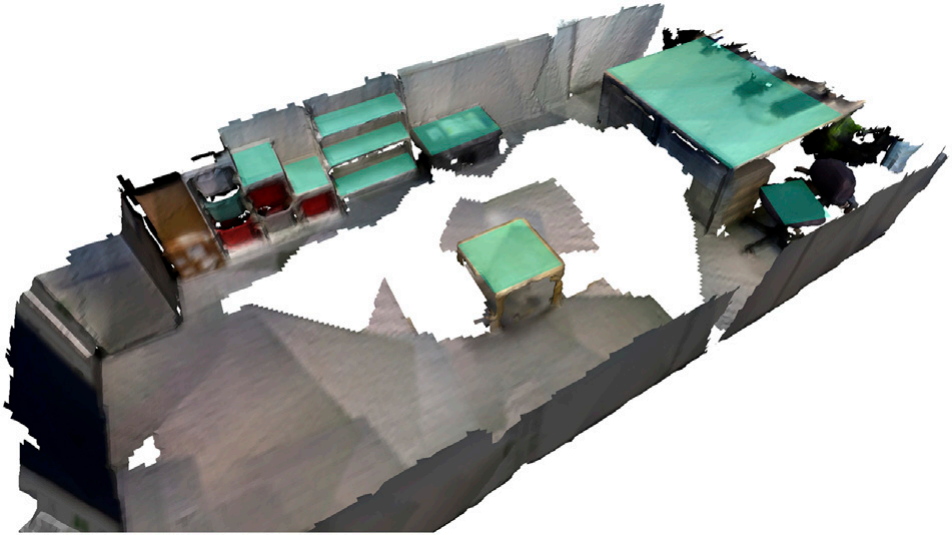
Reconstruction of the room used for the real robot experiments. The detected planes are highlighted in turquoise.

For the real-world robot experiments we use a Toyota Human Support Robot in one room with nine surfaces and conduct three runs with different object changes. Figure 6 presents the reconstruction of the room using *Voxblox*. It also shows the surfaces that are automatically extracted by exploiting semantic labeling as outlined in Section 3.3.

To create the waypoints for each surface, robot locations are generated at a distance of 20 cm to the convex hull around the plane oriented towards the plane center and evenly distributed every 30 cm around the circumference. To create the reference map (used for all three runs), the robot visits each surface and stores the detected objects in the database. Twenty out of 21 objects placed on the extracted planes are detected. The object locations then change three times for the three runs at times *t*_1_ to *t*_3_.

Table 5 presents the results. For each run we give the numbers of the objects assigned to the four categories compared to the ground truth. Missed objects are those that were not detected at all and therefore result in incorrectly categorized objects. The table also shows the number of false positive objects as defined in Section 4.1.2. The difference in the numbers of the last column between our approach and the ground truth stems from the sum of objects that were not detected (*#Missed*) and from wrongly matched objects. In summary, most of the objects are detected (65 of 70) and correctly classified (61 of 65). Including the removed objects, 65 of 74 objects are correctly categorized (last column in the table). For each run the detection and matching part took approximately 10 min on a standard laptop.

**TABLE 5:** Results of matching objects from the robot experiments. The rows with GT refer to ground truth for the respective runs 1 to 3.

Run	#Static	#Moved	#Removed	#Novel	#Missed	#FPs	#Correct
1	17	2	1	4	1	0	24
GT	18	2	1	4	0	0	25
2	12	5	2	3	1	0	22
GT	13	6	2	3	0	0	24
3	5	10	1	3	3	2	19
GT	8	12	1	4	0	0	25

**FIGURE 7:**
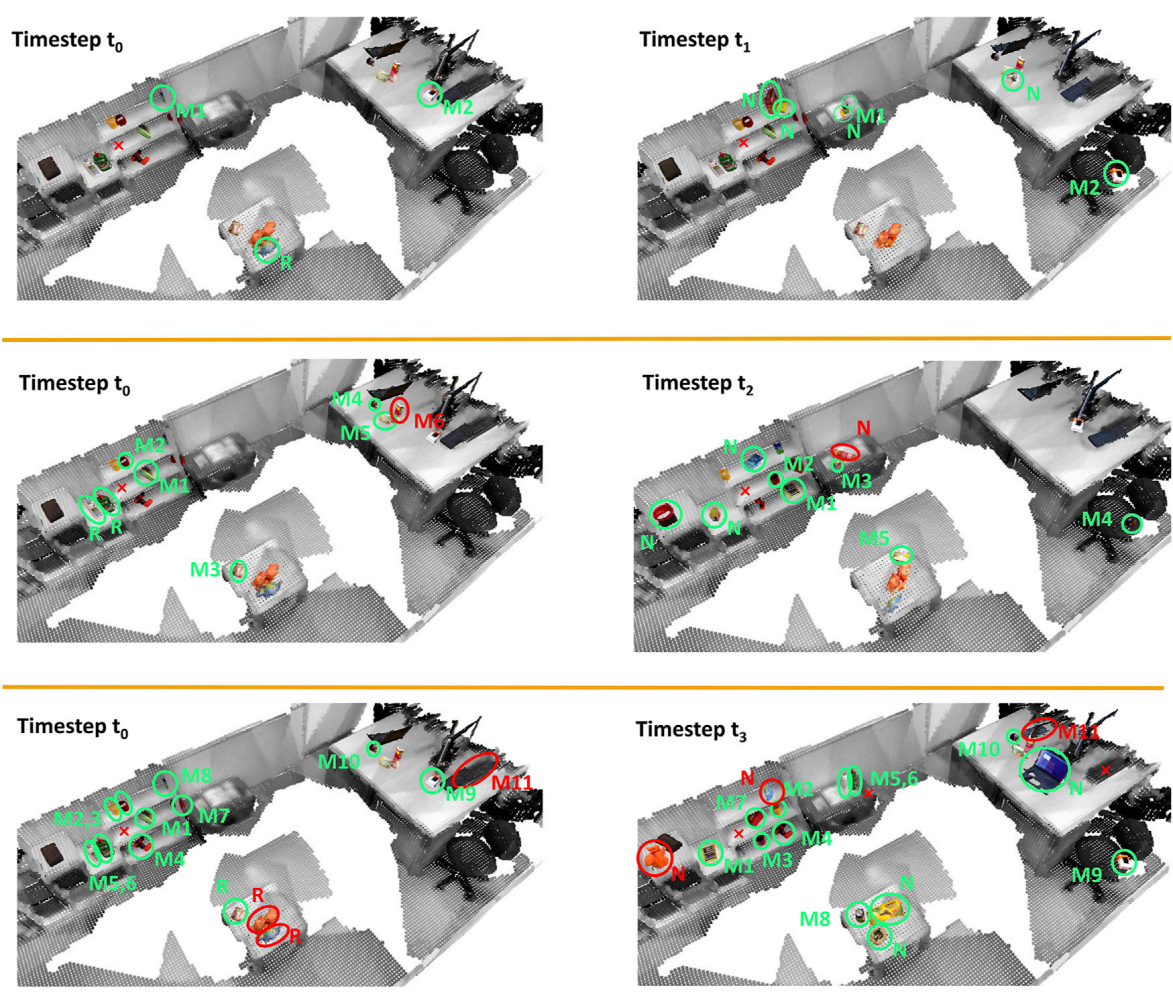
Results of real-world robot experiments. Each row shows the comparison of the reference room (first column) with the state of the same room at a different time (second column). Each detected object is marked with an ellipse and labeled with M (moved), R (removed), or N (novel). Correct categorizations are colored in green, wrong ones in red. Objects, which were matched, have the same number assigned. For simplicity static objects are not shown and missed objects are marked with a red cross.

Figure 7 gives details about the matches. In run 1 all objects are matched correctly. In run 2, the Pringles can is misclassified when comparing the room at time *t*_0_ and *t*_2_. At *t*_0_ the Pringles can is close to a tea box, which is correctly categorized as moved, but region growing fails to stop at the object boundaries. Therefore, the two objects are assigned the same label. This leads to the error that the Pringles can at *t*_2_ is wrongly labeled as new. At time *t*_3_ the keyboard is partially occluded when the robot moves around the table. Consequently the reconstruction is only a planar surface, which is not considered as an object. As a consequence the keyboard from time *t*_0_ is matched to the keyboard of a laptop (labeled as M11 in Figure 7). The other wrongly categorized objects result from the inability to split the orange and blue object on the table in the middle of the room. Although for both objects PPF found the correct match and the confidence is the highest compared to other possible matches, it is still too low to accept the match. The reason is the low geometric overlap because the two objects are only partially reconstructed at *t*_3_ but almost a full model exists from *t*_0_.

### 4.3 Discussion

Based on the evaluation results using ObChange and the robot experiments, in the following sections we highlight the findings on remaining open challenges due to factors such as robot localisation, covering surfaces with views, the detection of small objects, partially occluded objects, and quality of reconstructions.

#### 4.3.1 Robot Localization Error

Precise robot localization is necessary for most change detection applications. It supports the pairing of frames as well as the creation of clean reconstructions. In our experience the performance of dense visual SLAM methods in environments with areas of little visual and geometric features greatly benefit from integrating odometry data assuming state-of-the-art localization. Our solution to integrate odometry data to the *ElasticFusion* framework could reconstruct all surfaces in *ObChange* because the robot drift is insignificant. In case this cannot be guaranteed, the recent work by Houseago et al. (Houseago et al., 2019) shows how to integrate odometry data and dense visual SLAM in the presence of drift. Although their method could be integrated in our system, we opted for a computationally cheap approach. This is important when working with mobile robots, where limited resources have to be shared among different system components.

#### 4.3.2 Search Space

**FIGURE 8:**
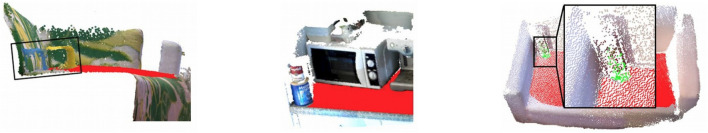
Examples of missed objects. Extracted planes are visualized in red. First image: two objects in black rectangle. Only the yellow cup is detected because the couch seat is too curved. Second and third image: only few object points are within the convex hull of the plane and therefore classified as noise and rejected. Note, that in the third example the reconstruction is very sparse because the robot did not get close enough to the surface.

Detecting objects is often a trade-off between reducing the search space and not missing objects (examples are shown in Figure 8), while at the same time the chance to detect false positives increases with a less restricted search space. The concept of surfaces reduces the search space significantly but may lead to missed objects if the surface is occluded or if it is not planar. We decrease the number of false positive detections by utilizing a shrinked convex hull of the surface, but this may cause objects at the border of a surface to not be detected.

#### 4.3.3 Detection of Small Objects

The detection of small objects is a well-known problem. Especially when working with 3D maps, actions taken to reduce the effect of noise and misalignments are the reason for filtering small objects. Our evaluation shows that neither the learning-based approaches nor the surface concept approach are capable of reliably detecting small objects.

#### 4.3.4 Occlusion

**FIGURE 9:**
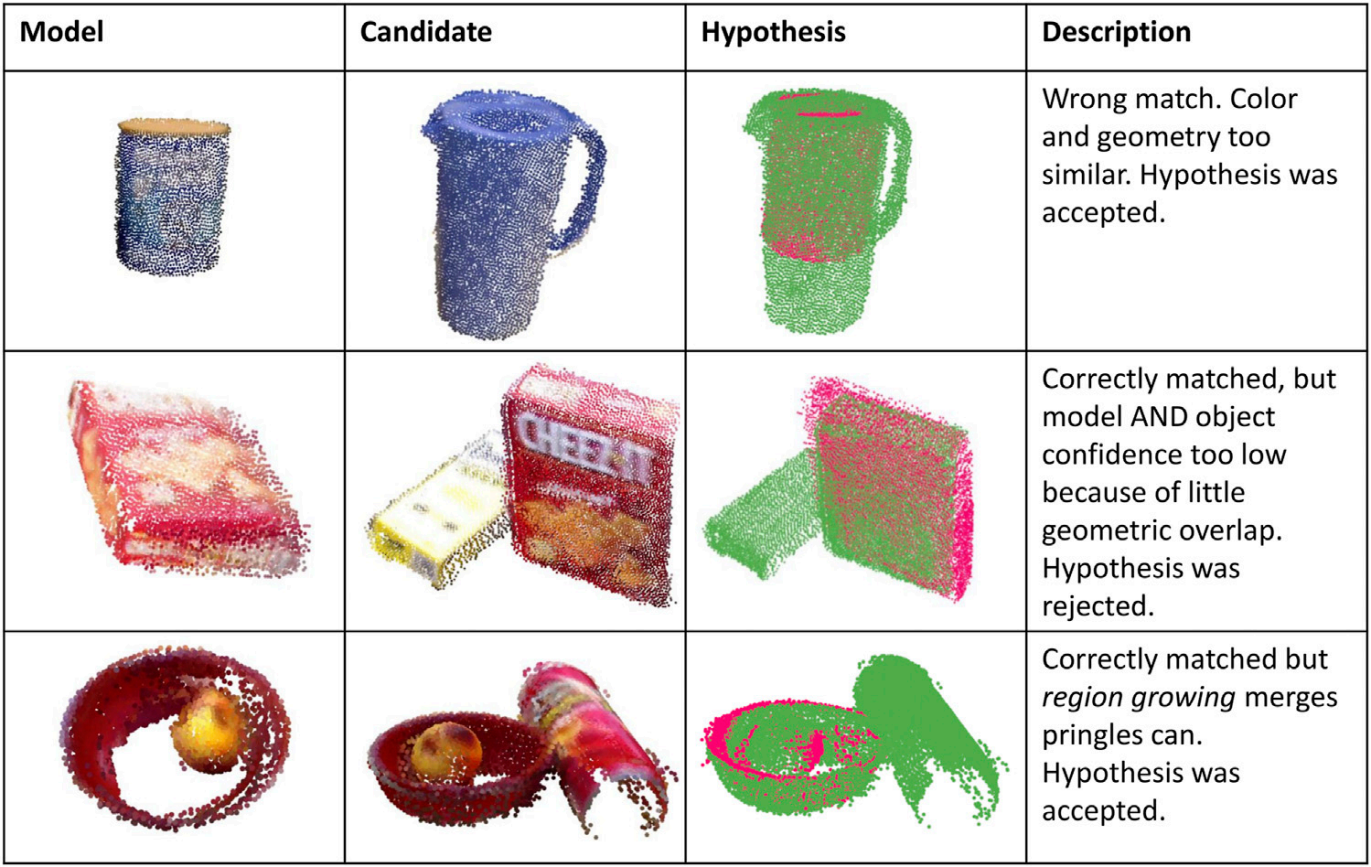
Examples of hypotheses generated based on PPF.

Occlusion poses a significant challenge for object matching because parts of an object are missing. The same accounts for incomplete reconstruction due to view limitations. Generally, ambiguities due to truly similar object appearance or partial reconstructions (see Figure 9 first two examples) need to be counter-measured by taking into account as much information as possible, for example, color PPF (Choi and Christensen, 2012) could provide better a priori hypotheses at the cost of higher computational efforts.

#### 4.3.5 Object Matching Verification

A verification step to check if a match is physically plausible, similar to Furrer et al. (2018) or Bauer et al. (2022), could help in some cases to reject inconsistent matches. However, this method of verification does not work in all cases; approaches often fail when objects are partially visible or symmetric. For example, a tube was detected at *t*_0_ (*tube*_0_) and at *t*_1_ (*tube*_1_), but was only half visible in the latter. The object matching and registration is ambiguous and resulted in aligning *tube*_1_ to the top of *tube*_0_. Projecting *tube*_0_ into the *t*_1_-recording resulted in half of the tube being below the supporting plane, which is physically implausible. This triggered a rejection even though the matched objects are the same. More generally, the fact that detected objects can actually be a heap poses challenges in geometric and semantic verification.

#### 4.3.6 Reconstruction Quality

Clearly, the quality of the surface reconstruction and therefore the 3D object models impacts the performance of object matching applications. While *ElasticFusion* tends to create reconstructions with smoothed normals, it also connects spatially close objects with additional points having a continuous color gradient. While smooth reconstructions are appealing and useful for many applications, we would rather prefer sharp reconstructions, especially when it comes to processing steps such as matching or region growing which favor distinctive geometric features.

## 5 Conclusion

In this paper we tackled the core perception capabilities for open-world operation in the context of the object mapping task. This was defined as comparing the locations of objects in the present visit (present object map) to a previously stored reference map. Without loss of generality, we proposed to create a 3D reference map of the environment. Comparing complete room reconstructions is impractical for many application scenarios; therefore, we presented a concept where only local surfaces are reconstructed. This has the benefit that local comparisons are performed more rapidly and that these local planes are reconstructed more accurately. The latter is critical to enhance object detection and matching results. We showed that semantic segmentation methods are suitable to autonomously provide a high-level partitioning into relevant horizontal surfaces.

The key step of object mapping is to compare object detections and match objects from two different time instances. A main contribution of this work is the perception of all possible cases of static, moved, removed, and novel objects. To evaluate the novel approach, we compared against two baselines using state-of-the-art methods. For a quantitative evaluation we presented the *ObChange* dataset consisting of five different rooms, an extension of the dataset in Langer et al. (2020), for the object mapping task. Results indicated that the proposed method significantly improved over the baselines in terms of detected objects as well as the accuracy of categorization. Furthermore, we conducted real-world experiments with an autonomous mobile robot to demonstrate our developed capabilities in a realistic setting. The experiments and evaluation give clear hints on the remaining open challenges such as improving the reconstruction quality in terms of accuracy as well as completeness. Another open challenge is the detection of small objects and objects that are partially hidden, where in both cases multiple views and using cues that there might be another object and creating a close-up view might be a future solution to work on.

Future work is also needed to realize the application scenarios such as tidy up. While we focused on the key aspect of perceiving all object changes, object pose estimation needs to be further integrated with grasping. Extending the reference map to a knowledge base may help to keep track of where objects have been seen in the past and might be found when requested by the user (Tenorth and Beetz, 2013). Over time, the more changes happen, the better the environment is segmented and explained. Finally, given we can detect novel objects, it would be beneficial to merge partially detected objects into a complete 3D model as proposed in Furrer et al. (2018).

## Data Availability

The dataset generated for this study is available at https://doi.org/10.48436/y3ggy-hxp10.

## Footnotes

1
http://juxi.net/challenge/tidy-up-my-room/
